# Diversification and Evolution of Vancomycin-Resistant Enterococcus faecium during Intestinal Domination

**DOI:** 10.1128/IAI.00102-19

**Published:** 2019-06-20

**Authors:** Krista A. Dubin, Deepti Mathur, Peter T. McKenney, Bradford P. Taylor, Eric R. Littmann, Jonathan U. Peled, Marcel R. M. van den Brink, Ying Taur, Eric G. Pamer, Joao B. Xavier

**Affiliations:** a Immunology Program, Sloan Kettering Institute, Memorial Sloan Kettering Cancer Center, New York, New York, USA; b Computational and Systems Biology Program, Sloan Kettering Institute, Memorial Sloan Kettering Cancer Center, New York, New York, USA; c Center for Microbes, Inflammation and Cancer, Sloan Kettering Institute, Memorial Sloan Kettering Cancer Center, New York, New York, USA; d Adult Bone Marrow Transplantation Service, Memorial Hospital, Memorial Sloan Kettering Cancer Center and Weill Cornell Medical College, New York, New York, USA; e Infectious Diseases Service, Memorial Hospital, Memorial Sloan Kettering Cancer Center, New York, New York, USA; University of California, Davis

**Keywords:** bacterial evolution

## Abstract

Vancomycin-resistant Enterococcus faecium (VRE) is a leading cause of hospital-acquired infections. This is particularly true in immunocompromised patients, where the damage to the microbiota caused by antibiotics can lead to VRE domination of the intestine, increasing a patient’s risk for bloodstream infection. In previous studies we observed that the intestinal domination by VRE of patients hospitalized to receive allogeneic bone marrow transplantation can persist for weeks, but little is known about subspecies diversification and evolution during prolonged domination. Here we combined a longitudinal analysis of patient data and *in vivo* experiments to reveal previously unappreciated subspecies dynamics during VRE domination that appeared to be stable from 16S rRNA microbiota analyses. Whole-genome sequencing of isolates obtained from sequential stool samples provided by VRE-dominated patients revealed an unanticipated level of VRE population complexity that evolved over time. In experiments with ampicillin-treated mice colonized with a single CFU, VRE rapidly diversified and expanded into distinct lineages that competed for dominance. Mathematical modeling shows that *in vivo* evolution follows mostly a parabolic fitness landscape, where each new mutation provides diminishing returns and, in the setting of continuous ampicillin treatment, reveals a fitness advantage for mutations in penicillin-binding protein 5 (*pbp5*) that increase resistance to ampicillin. Our results reveal the rapid diversification of host-colonizing VRE populations, with implications for epidemiologic tracking of in-hospital VRE transmission and susceptibility to antibiotic treatment.

## Abstract (precis)

Vancomycin-resistant Enterococcus faecium (VRE) is a leading cause of hospital-acquired infections. This is particularly true in immunocompromised patients, where the damage to the microbiota caused by antibiotics can lead to VRE domination of the intestine, increasing a patient’s risk for bloodstream infection.

## INTRODUCTION

A healthy intestinal tract contains a multitude of bacteria from diverse species, collectively referred to as the intestinal microbiota (1–3). Immunocompromised patients, such as those undergoing allogeneic hematopoietic cell transplantation (allo-HCT), receive broad-spectrum antibiotics to prevent and treat infections, in the process losing large portions of their intestinal commensal bacteria (4–6). In the setting of antibiotic-mediated microbiota destruction, vancomycin-resistant enterococci can rapidly expand to high densities in the gastrointestinal tract, leading to intestinal domination that persists for days after the cessation of antibiotic administration. In animal models of microbiota injury, mice remain susceptible to domination by vancomycin-resistant enterococci for over 4 weeks after ampicillin treatment; once vancomycin-resistant enterococci dominate the intestine, they can persist at a high density for months. In allo-HCT patients, vancomycin-resistant enterococcus domination of the gastrointestinal tract raises the risk of bacteremia (6) and the risk of graft-versus-host disease (7–9).

Intestinal bacteria can evolve rapidly in the intestinal tract of mammalian hosts; in some settings strains with higher antibiotic resistances can emerge (10–12). While the majority of vancomycin-resistant enterococci that cause bacteremia are classified as Enterococcus faecium (77%), this species consists of many strains that differ in antimicrobial resistance (13). Intestinal colonization by E. faecium and the associated excretion of large numbers of viable bacteria in the health care setting are major contributors to patient-to-patient transmission (14). A patient may be colonized by multiple E. faecium strains, and each strain could accumulate new mutations, forming a dynamic multistrain population, which complicates antibiotic treatment (15). Patients already colonized with a vancomycin-resistant enterococcus strain can even acquire another strain from the hospital, which adds another source of subspecies diversity and can lead to recurrent bacteremia (16). The E. faecium dominations that we have described (6), which appear to be stable over time when characterized by 16S rRNA gene amplicon sequencing, may hide underappreciated subspecies diversity and *in vivo* dynamics. Such dynamics of subspecies diversity could confound epidemiological studies attempting to track antibiotic-resistant infections and contribute to increasing levels of antibiotic resistance.

Here, we demonstrate that the vancomycin-resistant E. faecium (VRE) populations dominating the gastrointestinal tract of allo-HCT patients are indeed dynamic and undergo day-to-day diversification. Intestinal colonization of mice by a single bacterium revealed the rapid evolution of VRE and the generation of multiple parallel lineages that competed in the gut lumen. Using a longitudinal approach, we show that fitness differences between VRE clonal subpopulations create competition dynamics that guide evolution of the overall population, and mathematical models of *in vivo* evolution provide insights into the shape of the evolutionary fitness landscape. Our results have implications for the epidemiology of VRE infections in health care settings and suggest that tracking pathogen evolution within patients by deeper and broader sequencing of clinical samples and isolates can provide clinically useful information.

## RESULTS

### Genomic diversity of VRE in the gut of an individual patient.

**FIG 1:**
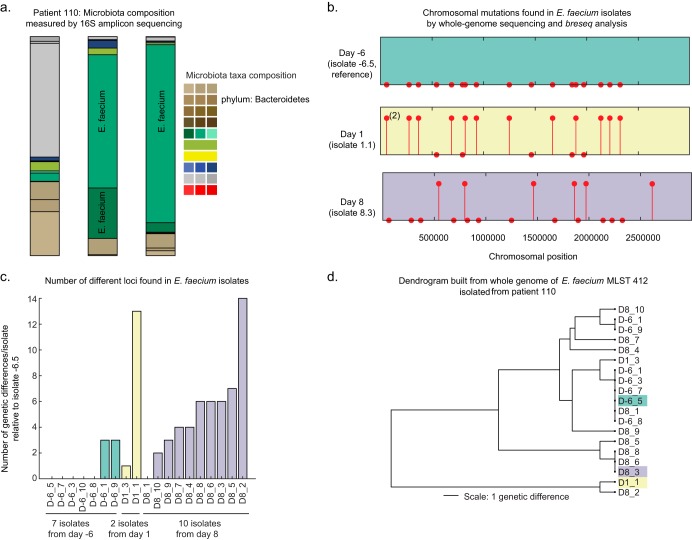
Within-host diversity of a single allo-HCT patient at MSKCC within and across time. (a) The relative abundances of microbiota components measured by 16S rRNA sequencing and classified at the species level are shown for 3 stool samples collected. Days are relative to the time of HCT. (b) Lollipops indicate the presence of an SNV or a short indel along the chromosome for 3 VRE isolates taken on days −6, 1, and 8. (Markers without stems denote positions of mutations found in other genomes.) (c) Bar chart of the number of variants per isolate on days −6, 1, and 8. (d) A dendrogram of the MLST 412 isolates cultured across days −6, 1, and 8 based on whole-genome comparisons using the PATRIC web portal. Branch lengths are proportional to the number of genetic differences.

We first characterized the cross-sectional diversity of vancomycin-resistant E. faecium (VRE) in an allo-HCT patient, herein identified as patient 110 (pt110) (Fig. 1a). Microbiota analysis by 16S rRNA gene amplicon sequencing showed that the patient was initially colonized with *Enterococcus* at a <10% relative abundance 6 days prior to allo-HCT (day −6), after which *Enterococcus* expanded and remained at a high density in fecal samples. To determine the complexity of the patient’s *Enterococcus* domination, we initially cultured and whole-genome sequenced 3 VRE isolates from fecal samples, one each from days −6, 1, and 8. We selected the genome of an isolate from day −6, classified as E. faecium, as the reference genome, and we aligned sequence reads from the day 1 and 8 VRE isolates using analysis with the *breseq* tool to identify genetic differences, generally called variants, which included single-nucleotide variants (SNVs) as well as small insertions-deletions (indels). The genomes of isolates from days 1 and 8 were highly similar to the genome of the day −6 isolate, but they contained unique variants (Fig. 1b), suggesting that these VRE variants might have arisen independently from an ancestral strain prior to day −6.

**FIG 2:**
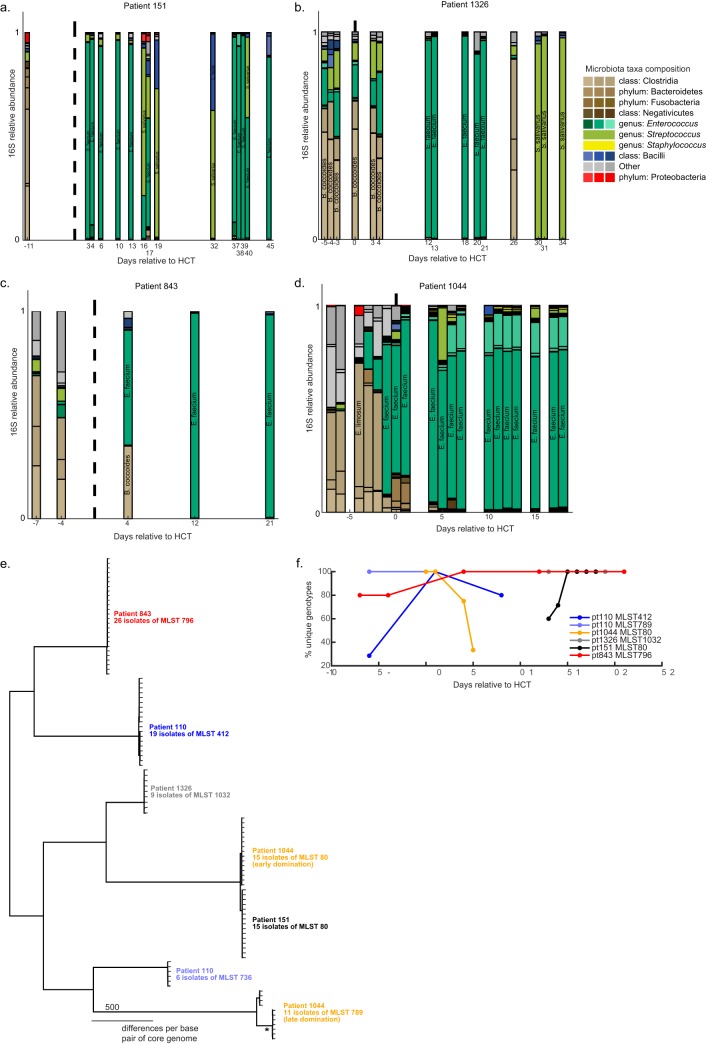
Unique patterns of genetic variation are detected on each day for VRE-colonized allo-HCT patients and are acquired at variable rates. (a to d) The relative abundances of microbiota components (amplicon sequence variants [ASVs]) measured by 16S rRNA sequencing and classified at the species level are shown for 4 patients. Days are relative to the time of HCT. The vertical dotted black line denotes the day of cell infusion. S. salivarius, Streptococcus salivarius; L. lactis, Lactococcus lactis; B. coccoides, Blautia coccoides; E. limosum, Eubacterium limosum. (e) Multiple VRE isolates were cultured from each sample from 5 additional allo-HCT patients and sequenced by use of the Illumina MiSeq platform. *De novo* assemblies were constructed from Illumina reads using the SPAdes genome assembler. Core genome alignments were obtained using the Roary program, and a maximum likelihood tree was assembled using the FastTree program. (f) One isolate per patient was sequenced by use of both the Illumina MiSeq and Nanopore MinION platforms. A hybrid assembly was generated with the Unicycler program, and variants were called and corrected with the tools *breseq* and bcftools. Reads for all patient isolates were aligned to their own reference sequence, and the *breseq* tool was used to detect mutations. The proportion of new unique genotypes observed per day was plotted for each day. The reference strain (which contained no variation, by definition) was not counted as a unique genotype.

We analyzed an additional 22 VRE isolates from this patient from days −6, 1, and 8. Using the short reads, we assembled draft genomes for each of the 25 isolates, and we analyzed the assembled contigs. The analysis revealed that pt110 harbored two distinct VRE strains: 19 isolates belonged to the multilocus sequence typing (MLST) 412 strain, and 6 isolates belonged to a less frequent MLST 736 strain (Fig. 2e). To gain more insight into the substrain diversification of VRE, we first focused on the predominant MLST 412 strain. We compared the genomes of the 19 isolates, where 7 isolates came from day −6, 2 came from day 1, and 10 came from day 8. As the *Enterococcus* population increased in abundance between days −6 and 8, reaching over 90% of the microbiota composition, as determined by 16S rRNA sequencing, the numbers of genetic variants detected increased over time (Fig. 1c). A dendrogram of the 19 isolates from MLST 412 based on their core genome indicated that the genetic differences did not accumulate in a constant proportion across the passage of time (Fig. 1d).

### Additional allo-HCT patients with *Enterococcus* dominations reveal hidden dynamics of subspecies diversity.

To broaden our analyses of subspecies diversification of VRE within patients, we selected 4 additional patients in whom intestinal domination by *Enterococcus* was observed, as determined by 16S rRNA gene amplicon sequencing (Fig. 2a to d). We isolated individual VRE colonies from banked fecal samples and genome sequenced a total of 101 isolates, which confirmed that the colonizing species was E. faecium in all patients (Fig. 2e). Similar to pt110, pt1044 was cocolonized with two distinct VRE strains, with MLST 80 predominating initially, followed by MLST 789 at later time points. For pt1044, one strain replaced the other and went on to dominate the patient’s microbiota. Interestingly, each VRE strain in the patients demonstrated substrain diversity and dynamics (Fig. 2a to e).

To better characterize the pattern of intrapatient VRE diversity, we applied a similar longitudinal analysis to the five patients, where we identified novel variants (SNVs and indels) from multiple isolates from each patient by comparing the genomes of the isolates to a reference genome generated from one of the earliest isolates obtained from each patient (see Fig. S1 in the supplemental material). As with pt110, domination by VRE was associated with a diversity of variants that changed in abundance over time, and as in pt110, the pattern of new variants that emerged did not reflect the passage of time. To quantify VRE diversification, we determined the number of new variants among isolates obtained from the same patient and belonging to the same MLST and plotted the percentage of unique genotypes (determined as the number of genotypes not previously seen per 100 total genotypes) for each patient. The subspecies population dynamics varied between patients, indicating that the path of VRE evolution in patients is likely not deterministic (Fig. 2f). The isolates of pt843 and the MLST 412 isolates of pt110 showed an increase in the proportion of new genotypes detected postexpansion. The MLST 80 isolates of pt1142 had a collapse in their population diversity, reverting to the reference strain, as no isolates from this MLST harbored variants at the final time point (day 5). We investigated recurrently mutated genes for signatures of selection based on the ratio of nonsynonymous substitutions to synonymous substitutions (*dN*/*dS*) (Table S1). However, we found no significant signatures, possibly due to high variability and few recurrent mutations in patient isolates. We also analyzed the number of unique clones per day using generalized linear regression models to test if there were common patterns across patients (not shown). The high variability even within a patient from day to day prevented us from making any conclusions from this analysis. Taken together, these analyses suggest complex dynamics of VRE diversification in the gut similar to those observed with other gut bacteria (10), prompting further investigation.

### *In vivo* experimental evolution in a mouse model reveals rapid diversification.

**FIG 3:**
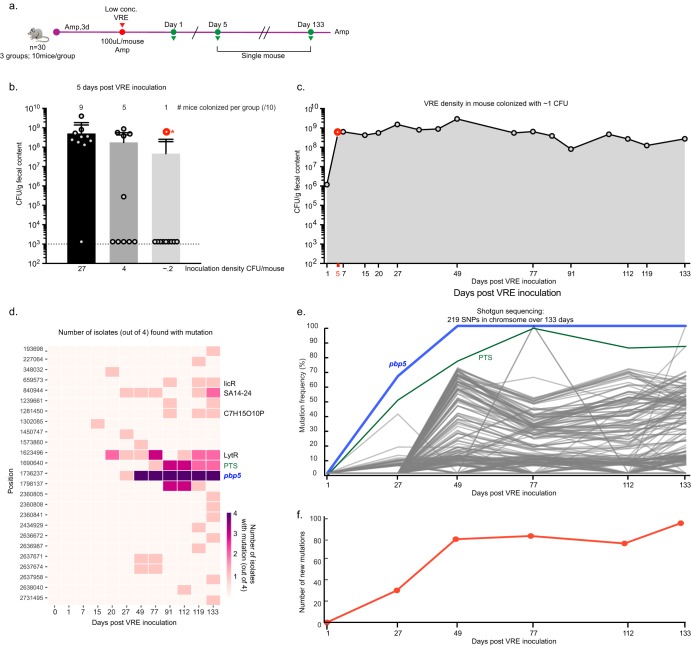
VRE expanded rapidly in the gastrointestinal tract after acquiring a nonsynonymous mutation in the gene encoding penicillin-binding protein 5 (*pbp5*). (a) Diagram of experimental design. An initial cohort of 30 mice was split into 3 groups, each of which was given a different number of CFU, and then housed singly. One mouse with the lowest level of inoculation was followed for 133 days. All mice were kept on ampicillin (Amp) for the duration of the experiment. 3d, 3 days. (b) Low inocula of VRE (number of CFU per mouse) were gavaged into ampicillin-treated mice. The height of the bars depicts the mean number of CFU per gram of fecal content for each condition. The number of mice that were successfully colonized per inoculated group by day 5 is plotted; data are for 10 mice per experimental group. The dose of 0.2 CFU/mouse, which is equivalent to 2 CFU/ml, infected 1 mouse out of 10 (circled in red). (c) This individual infected mouse was followed for a total of 133 days. The density of VRE was measured by plating fecal pellets on selective media. Four isolates were cultured from the fecal content obtained at the 11 time points indicated. (d) A heat map of the 25 mutations acquired in the chromosome across the 44 isolates. The *pbp5* mutation is indicated, as are other loci mutated in 3 or more isolates. (e) Frequency traces are shown for the 219 chromosomal mutations picked up by shotgun sequencing of fecal pellets from a single colonized mouse. SNPs, single-nucleotide polymorphisms. (f) Total number of mutations that achieved a ≥5% frequency in the shotgun sequencing data per day over the course of the experiment.

The deep analysis of VRE dominations in allo-HCT patients, which had previously appeared to be stable from 16S rRNA microbiota analysis, revealed unexpected dynamics of subspecies diversity. From these data, however, we could not determine precisely how long each patient had harbored VRE in the gut, and without this knowledge, we could not determine whether diversity developed after initial colonization, whether patients were initially infected with a complex VRE population, or whether patients kept acquiring new diverse clones during hospitalization. Therefore, we turned to a mouse model where we could monitor the expansion and diversification of VRE more precisely. We used a limiting-dilution experiment to colonize a mouse with ∼1 bacterial cell to study the dynamics of intestinal colonization starting from a monoclonal inoculum. Naive C57BL/6J mice, 8 to 10 weeks old, were all treated with ampicillin in their drinking water for 3 days, after which they were orally inoculated with a range of VRE concentrations (Fig. 3a). Among a group of 10 individually housed mice inoculated with 0.2 CFU of VRE per mouse, only 1 became colonized (Fig. 3b), strongly suggesting that this mouse was colonized by a single bacterium (from a simple calculation based on the Poisson distribution, the probability that the mouse received more than one bacterium was 0.0011). This mouse was continuously treated with ampicillin, and fecal samples were collected for 133 days to investigate the evolution of the colonizing VRE population (Fig. 3a).

The density of VRE detected in fecal samples stabilized at 5 days postinoculation (Fig. 3c). We obtained 4 VRE isolates at each of 11 time points between 1 and 133 days of intestinal colonization (Fig. 3c) and performed whole-genome sequencing to identify new genetic variants. The first mutation was detected in an isolate obtained at day 15 of colonization (Fig. 3d) and was followed by a rapid increase. By 49 days of colonization, we detected levels of VRE diversity within a mouse colonized with a single bacterium that approximated the levels of VRE diversity detected in the patients (Fig. 3d; Table S2).

To more completely analyze the diversity in the intestinal VRE population, we broadened our investigation by shotgun metagenomics sequencing of DNA extracted from fecal samples. We observed the expansion of many genetic variants, only a subset of which coincided with those sequenced from isolates obtained from the same mouse (Fig. 3e). The total number of genetic variants in the population stabilized after about 49 days, at a value of ∼80 variants, a surprisingly low number given the sheer number of possible genetic variants that could theoretically occur in the E. faecium genome (Fig. 3f). As observed with pt110, some mutations stabilized at high or low frequencies, while others disappeared from the population over time (Fig. 3e). Noticeably, a nonsynonymous mutation in the gene for penicillin-binding protein 5 (*pbp5*) became fixed in the population by day 49. This A→C *pbp5* mutation occurred in codon 434, changing the negatively charged aspartate to a nonpolar alanine within the transpeptidase domain of PBP5. The PBP5 protein has been shown to confer ampicillin resistance, and mutations in its gene have been documented in clinical isolates (17–21).

Individual mutations detected by shotgun sequencing displayed various temporal patterns: while the frequency of some increased steadily and became fixed in the population, others stabilized at a lower frequency or even rose and fell over time (Fig. 3d). This prompted us to characterize this dynamic pattern of VRE diversification quantitatively using a mathematical model.

### Mathematical modeling recaptures evolutionary dynamics.

**FIG 4:**
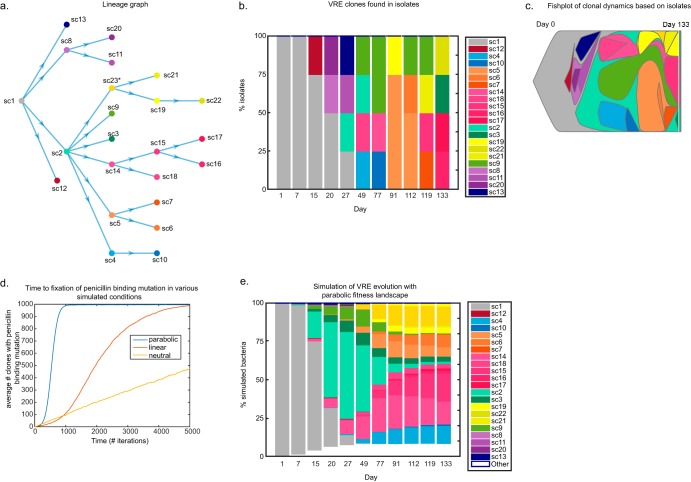
Sublineages coexist after rapid diversification, in which the *pbp5* mutation becomes fixed in the total intestinal VRE population. (a) A parsimonious graph of the 22 unique genotypes found among the 44 isolates. The branch of isolates harboring the *pbp5* mutation that evolved stems from sc2. The clone with an asterisk (sc23) was not experimentally observed but must have existed in order for the observed sc19, sc21, and sc22 genotypes to occur. (b) The frequency of each genotype found in the 4 isolates collected per day is plotted; related isolates are grouped into colored bins. (c) Clonal dynamics are captured in a fishplot. (d) The number of clones with a penicillin-binding protein mutation under the neutral, linear, and parabolic fitness landscapes is plotted over time. Each curve is an average for 50 independent *in silico* experiments. (e) Simulated VRE evolution under a parabolic fitness landscape, with a fitness boost given to experimentally observed genotypes with a *pbp5* mutation. Each genotype corresponds to a color according to the same scheme used in panel b, with the addition of white clones corresponding to genotypes that arose during the course of the simulation but that were not experimentally observed. One day corresponds to about 50 simulation iterations.

While metagenomic shotgun sequencing provided greater sampling depth, isolate sequencing from our *in vivo* model enabled us to link genetic variants within the same genome. Using this information, we classified “genotypes” as isolates that shared identical genetic variants, yielding a total of 22 VRE genotypes observed over the 133-day experiment. These genotypes were then used to assemble a lineage graph with maximum parsimony, depicting the relationships between the ancestral clone (sc1) and its evolved lineages (Fig. 4a). The grouping of genetic variants into genotypes also allowed us to depict the clonal evolution of VRE using isolate data alone (Fig. 4b and c) or combined isolate and shotgun sequencing data (Fig. S2a and b).

To understand the population dynamics governing VRE diversification, we generated an *in silico* simulation of our *in vivo* experiment. Our simulations began with a population made up of only the ancestral VRE clone, and each iteration of the simulation allowed random mutation at any of the 25 loci observed experimentally. With even just 25 loci, there were 2^25^, or 33,554,432, different possible clones, yet experimentally we observed only 22 clones. The purpose of the model was to test various fitness landscapes that guide this very large number of possible clones to the ones that were experimentally observed. We found that under neutral evolution the *pbp5* mutation did not fix in the population, unlike our experimental results (Fig. 4d). This clearly demonstrates that, in addition to random mutation, competition among clones due to variations in fitness likely shapes VRE diversification.

We therefore tested three possible fitness landscapes that may explain the data from our experiment: (i) linear, in which each additional mutation observed in our panel of sequenced isolates conferred additional fitness; (ii) logarithmic, in which additional mutations conferred increased fitness but with diminishing returns; and (iii) parabolic, in which few mutations conferred increased fitness but too many mutations resulted in a fitness cost (Fig. S2c). The linear and logarithmic fitness landscapes implicitly constrain genotypes observed later to be fitter than earlier genotypes. Additionally, we tested the possibility that the *pbp5* mutation may enhance fitness further still by adding the condition that clones harboring this mutation have higher fitness in the linear, logarithmic, and parabolic fitness landscapes, for a total of six conditions. These clones would follow the same fitness landscape pattern, but at a higher level (Fig. S2c). Importantly, our simulations operated under the assumption that experimentally observed clones fall on the fitness landscapes described above but that any of the other possible genotypes that were not isolated have a relative fitness that is much lower. Therefore, while they may arise in the course of random mutation, they are unlikely to persist in the population. Hence, our simulations were largely constrained to the clones depicted in the lineage graph (Fig. 4a).

Testing the above-described six conditions in our *in silico* experiments revealed that a parabolic fitness landscape where *pbp5* mutation-containing clones have higher fitness most accurately regenerated the population dynamics observed *in vivo*. This conclusion was based on both the rate of ancestral clone decline and the rate of expansion of *pbp5* mutant sublineages (hereby referred to as branch 2), as well as the time to fixation of this mutation in the population (Fig. 4d and e). A simulated linear fitness landscape with no additional fitness given to *pbp5* mutant clones also closely resembled the experimental data in terms of the expansion of branch 2 lineages but did not accurately reflect the experimentally determined time to fixation of the *pbp5* mutation in the population and the consequent outcompeting of other clones (Fig. 4d and Fig. S2d). Our simulations suggest that relative fitness differences between clones play an important role in shaping the evolution of VRE in the gut and posit that a parabolic fitness landscape—in which additional mutations initially confer increased fitness but beyond a certain point lead to a fitness cost—may govern the accumulation of genetic variants from the ancestral clone.

Since there is biological plausibility that the *pbp5* mutation would be advantageous, it is possible that the positive selection of clones with this mutation is the major force governing VRE evolution in our *in vivo* model. We therefore expanded our model to allow any clone with the *pbp5* mutation to have high fitness, even if such a clone was not experimentally observed among our isolates. To our surprise, we found that under a linear fitness landscape the results of the simulation almost never reflected the clones experimentally observed, due to accumulation of a number of genetic variants far greater than the number contained in our isolates (Fig. S2e). Furthermore, a parabolic fitness landscape only partially recaptured the clonal dynamics of the *in vivo* model (Fig. S2f). We hence surmise that if, indeed, any clone with the *pbp5* mutation occupies the same fitness landscape, the result of our experiment was just one of many rare events, and it is unlikely that we would have ever seen this rare event if the fitness landscape governing VRE evolution was linear and much more likely if it was parabolic. If constrained to the lineage graph, the parabolic fitness landscape consistently recaptures the population dynamics observed experimentally, as concluded above.

### Replicate *in vivo* experiments demonstrate the stochastic nature of within-host VRE evolution.

**FIG 5:**
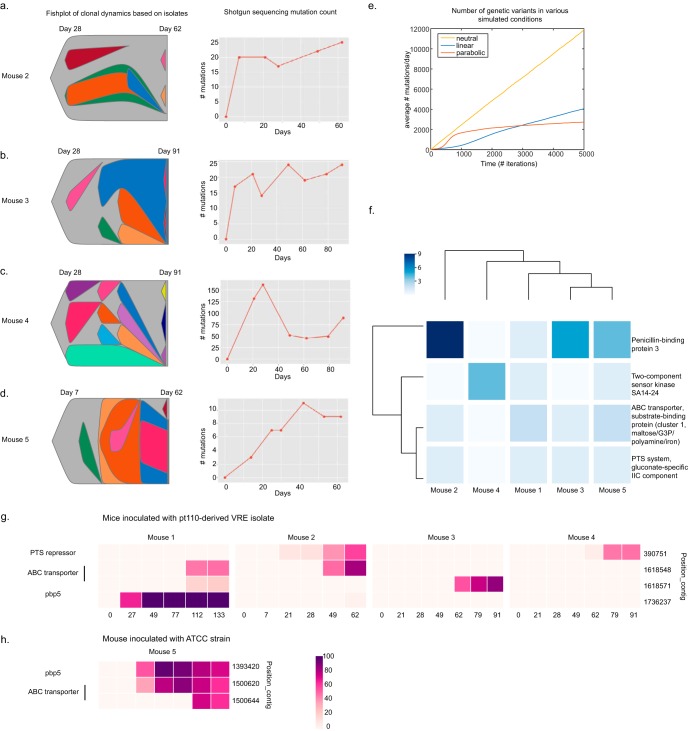
Within-host intestinal VRE evolution is a stochastic process. (a to d) (Left) Population dynamics are represented in fishplots based on combined isolate and shotgun sequencing data for mouse 2 (a), mouse 3 (b), mouse 4 (c), and mouse 5 (d). Mouse 5 was colonized by a laboratory E. faecium ATCC strain (ATCC 700221), while mice 2 to 4 were inoculated with a pt110-derived VRE isolate used in the experiment whose results are presented in Fig. 3. Bone marrow transplants with a 129S1/SvImJ mouse as the donor were performed on mouse 4 and mouse 5 prior to VRE infection. (Right) The total number of mutations that achieved a ≥5% frequency was calculated from the shotgun sequencing data. (e) The number of mutations under various simulation conditions (neutral, linear, and parabolic fitness landscapes) was plotted over time. (f) A list of genes mutated in ≥3 pt110 VRE isolate-colonized mice at a ≥1% frequency in the shotgun sequencing data was generated, and the subset that was also mutated at least once in the ATCC 700221-inoculated mouse is shown (*n* = 4). (Mouse 1 is the mouse from the experiment whose results are presented in Fig. 3.) The total number of mutations per gene within each mouse that appeared at any point during the experiment is represented in a heat map. The numbers at the top left report the total number of mutations for each gene summed across the mice. We analyzed 5 to 7 samples per mouse by shotgun sequencing. (g) Four mutations were found that were shared in ≥2 pt110 VRE isolate-colonized mice and that had reached a >20% frequency in at least one mouse at one time point in the shotgun sequencing data. Mutation frequencies over time are represented in a heat map for the 4 mice inoculated with the pt110-derived isolate. Note that for the *pbp5* mutation (locus 1736237), the maximum frequency reached in mouse 2 was 4%. (h) In the ATCC 700221-colonized mouse (mouse 5), 3 mutations were found in the ABC transporter and *pbp5* genes (no mutations occurred in the lactose PTS repressor gene).

The stochasticity of evolutionary processes means that replicate experiments often follow completely distinct paths (22). In order to ascertain how deterministic that the pattern of VRE diversification was, we repeated the above-described *in vivo* experiments four additional times. Using the same experimental setup of a 3-day ampicillin pretreatment and a low-dose inoculum, three mice received the VRE strain derived from pt110 (Fig. 5a to c) and one mouse was colonized with the ATCC 700221 VRE strain (Fig. 5d). In order to more closely approximate the conditions encountered by VRE strains colonizing allo-HCT patients, we sublethally irradiated two mice and transplanted major histocompatibility (MHC)-matched (minor antigen-disparate) allogeneic donor bone marrow prior to VRE inoculation (Fig. 5b and c).

Our results confirmed the stochastic nature of within-host VRE evolution because each mouse exhibited a unique pattern of VRE diversification (Fig. 5a to d, first column, and Fig. S3). The total number of genetic variants and the rate of their accumulation varied among the mice. Surprisingly, despite the differences in population dynamics, the population diversity reached a maximum for all mice, which agrees with our parabolic fitness model (Fig. 5a to d, second column; Fig. 5e; Fig. S4, columns 2 and 3).

Moderate- to high-frequency mutations in the *pbp5* gene were detected in two mice (mouse 3 and mouse 5), highlighting the positive selection of various penicillin-binding protein mutants in the setting of continuous ampicillin treatment (Fig. 5b and d). Importantly, similar to our finding with mouse 1, the evolved strains of these two mice outcompeted the ancestral strain (Fig. 5b and d and Fig. 4c). Interestingly, mouse 2 acquired *pbp5* mutations late in the course of the experiment, at day 62, and achieved a maximum frequency of only 10.5%, possibly because these variants may not have had sufficient time to exploit their fitness advantage.

Expansion of *pbp* mutations, however, did not occur in every host. The *pbp5* mutations did not become fixed in the VRE population in mice 3 and 5 (Fig. S4c and e). Interestingly, for both of these mice, we detected 3 unique *pbp5* mutation per mouse in the isolates; clonal interference among *pbp5*-evolved sublineages may have prevented the fixation of any one mutation.

The genetic variants that accumulated in our replicate *in vivo* experiments differed between mice, leaving open the possibility that minor differences in host environments cause divergent adaptations. A number of mutations in addition to *pbp5* reached high levels, suggesting alternative paths to improved fitness (Fig. 3e and Fig. S4a to e, first column).

To determine which genes might harbor a signature of positive selection, we identified 13 genes that were mutated in 3 or more of the 4 mice infected with the pt110-derived VRE strains. We then analyzed the subset of genes, 4 in total, that had also been mutated in the VRE ATCC 700221 strain-inoculated mouse. Here, we applied a lower-frequency detection threshold of 1%, as we were also interested in rarer mutations. The *pbp5* gene acquired 19 mutations across the 5 mice, and it was mutated over three times more frequently than the other 3 genes (Fig. 5f). As previously discussed, while mouse 2 acquired 11 unique *pbp5* mutations, none of them reached a high frequency (maximum, <11%). Mutations in the ABC transporter, SA14-24 serine kinase, and gluconate-specific phosphotransferase (PTS) system component genes suggest that changes in environmental information sensing/processing pathways may contribute to VRE adaptation in the gastrointestinal tract. Notably, the isolate data captured only the *pbp5* mutation across the mice in our cohort. As mentioned above, 3 mice had *pbp5* mutations that were detected in the isolate data, with an average of 2.3 SNVs per gene (Table S3). Mutations in the ABC transporter detected in the shotgun sequencing data were present only in the isolates from the ATCC 700221-inoculated mouse (mouse 5). Conversely, the SA14-24 serine kinase, gluconate PTS system component, and lactose PTS system repressor mutations were found in pt110-derived isolates. This may underscore that the medium used to isolate single clones from intestinal VRE populations may introduce biases (Fig. S4a to e, fourth column).

To explore this further, we examined the genetic variants in our shotgun sequencing data that occurred in 2 or more of the 4 mice inoculated with the pt110-derived VRE strain, out of the total of 2,672 variants detected within this data set that achieved a ≥1% frequency. We hypothesized that mutations conferring a fitness advantage could be identified by satisfying two conditions: (i) that they arise independently in *in vivo* experiments and (ii) that they achieve a substantial frequency (>20%) in at least one mouse at one or more time points. The frequency threshold of 20% was selected after visualizing the variant frequency data obtained from shotgun sequencing. Furthermore, we focused on mutations that could confer a functional change, either by a nonsynonymous amino acid change or, for intergenic mutations, by occurring within 300 bp of a translation start site (*n* = 781 functional SNVs in our data set). Out of 38 mutations shared by multiple mice, we found 4 that satisfied both conditions: the nonsynonymous *pbp5* SNV (locus 1736237), an intergenic SNV upstream of a lactose phosphotransferase (PTS) repressor (locus 390751, −181 bp upstream), and two mutations in an ABC transporter (maltose/G3P/polyamine/iron) (loci 1618548 and 1618571) (Fig. 5g). We sought to confirm if any of these mutations reached moderate frequencies (>20%) in the ATCC 700221-inoculated mouse. We detected one in the *pbp5* gene (locus 1393420) and two in the ABC transporter gene (loci 1500644 and 1393420) (Fig. 5h). Taken together, these data highlight the importance of the *pbp5* gene for intestinal VRE fitness in the presence of ampicillin and suggest other targets of positive selection.

### Parabolic fitness landscape detected in patient shotgun sequencing data.

**FIG 6:**
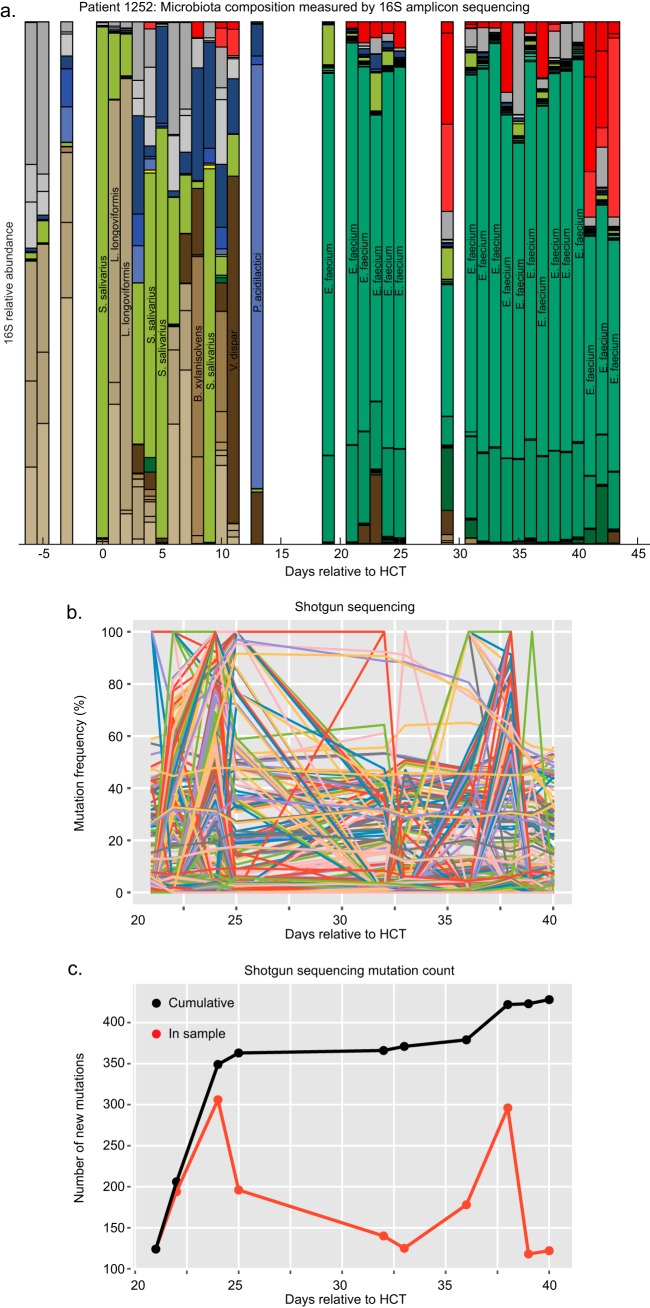
VRE diversification plateaus early in the intestinal domination of an allo-HCT patient. (a) The relative abundances of microbiota components measured by 16S rRNA sequencing and classified at the species level are plotted over the course of hospitalization for pt1252. Days are relative to the day of transplantation (the color key can be found in Fig. 1a). S. salivarius, Streptococcus salivarius; L. longoviformis, Lactonifactor longoviformis; B. xylanisolvens, Bacteroides xylanisolvens; V. dispar, Veillonella dispar; P. acidilactici, Pediococcus acidilactici. (b) Stool samples with >90% VRE domination were analyzed by high-throughput shotgun sequencing. The frequencies of all variants found along the 4 contigs that represent the chromosome in the pt1252 reference isolate are shown. (c) The number and cumulative number of variants achieving a ≥5% frequency per day are reported from the shotgun sequencing data.

To validate our findings in a patient, we shotgun sequenced the samples with >90% VRE domination banked from a sixth patient, pt1252, to better determine the frequency dynamics underlying the subspecies evolution (Fig. 6a). For pt1252, one VRE isolate (belonging to MLST 412) was cultured from the first day of known VRE domination (day 19) and used as a reference. Metagenomic binning of the reads obtained from shotgun sequencing revealed that one VRE strain (from MLST 412) dominated the gastrointestinal tract. Individual chromosomal variants displayed diverse dynamics over the course of VRE domination (Fig. 6b), a finding which looked similar to our *in vivo* experimental data (Fig. 3e and Fig. S4a to e). Some variants stabilized at high or low frequencies, while others disappeared from the population over time. The accumulation of unique genetic variants in the VRE metagenome of this patient plateaued, a shape expected under a parabolic fitness landscape (Fig. 6c, black line). However, population diversity appeared to rise and fall when calculating the total number of variants per day (Fig. 6c, red line). These data suggest a rapid diversification that resulted in 2 competing subclades, with the more distant of the two from the reference rising in proportion on days 24 and 38. However, the possibility that an alternative hypothesis, that these two subclades were acquired together, cannot be excluded from these data. Together, this pattern of VRE diversification fits with our *in vivo* experimental data and highlights the selective pressures of a complex antibiotic treatment regimen.

## DISCUSSION

Our survey of intestinal VRE in patients revealed not only previously underappreciated diversity within single strains but also that such diversity rapidly changes over time. Using a combination of clinical, experimental, and mathematical approaches, we characterized population dynamics and revealed evidence of a general fitness landscape that could shape VRE evolution *in vivo*. Furthermore, sustained antibiotic pressure likely led to the positive selection of *pbp5* mutations, indicated by their presence in four out of five mice following low-inoculum colonization with VRE.

Our finding that the *Enterococcus* dominations apparently stable at the level of 16S rRNA-based classification can obscure rich subspecies dynamics raises interesting questions. First, from our initial mouse experiment, it is curious that even after gaining the *pbp5* mutation, clones continued to evolve and obtained additional mutations that allowed them to outcompete clones with the *pbp5* mutation alone. This suggests that the *pbp5* mutation was not the sole force guiding VRE evolution. It is possible that the mutations compiled on top of *pbp5* have a biological mechanism that increased fitness in the presence of an antibiotic, or it is possible that diversity in and of itself was advantageous. For example, one of the mice that presented *pbp5* mutations also had a mutation in a gluconate-specific IIC component, responsible for carbohydrate transport in bacteria, which persisted at a higher frequency than *pbp5* and fixed in the population (mouse 3). VRE with this mutation could have higher sugar uptake and therefore a metabolic fitness advantage; it will be interesting to study how other modes of fitness gains interact with antibiotic resistance. Second, it is interesting that the total number of genetic variants observed at any given time seemed to stabilize rather than continue to increase linearly. We speculate that deleterious mutations and negative epistasis might keep subspecies diversity in check, despite the large population sizes and relatively high mutation rates of these bacterial populations. Third, our results showed many more mutations in this species compared with what has previously been observed in other species evolving in the intestine of mice (23, 24). The reason for this discrepancy remains unclear, but as more data on the evolution of gut bacteria *in vivo* become available, it will be interesting to compare how the rates of adaptation differ across taxa, a finding that is crucial both to our basic understanding of gut microbiome biology and to our understanding of the evolution of antimicrobial resistance in enteric pathogens.

Our findings recall the diminishing returns epistasis or negative epistasis in Escherichia coli studies, which contribute to decreasing rates of fitness gains during adaptation (25, 26). An important distinction is that these studies only considered mutations shown to have beneficial effects, while our model does not have this constraint. Therefore, we model fitness landscapes that not only have diminishing but positive gains in fitness (log landscape) but also decreased fitness at a high mutational load (parabolic landscape).

We chose simple approaches whenever possible, because the simplest approaches sufficed to make our main point that VRE evolution *in vivo* is underappreciated. First, we chose a method for calling mutations from metagenomic sequencing that relied on simplifying assumptions. Distinguishing error from true mutations is a notoriously difficult task, and detection of variants that occur at a low frequency, such as 1%, is especially prone to false positives. Improved methods could use a two-step mutation-calling protocol that would first identify polymorphisms and then look for support in each sample from a patient or mouse. This alternative can potentially improve the calculation of genotypes observed at any given time point and the comparison to evolutionary simulations. Still, this is not a key issue for the overall result that there is substantial change that would be inaccessible by typical 16S rRNA gene amplicon analysis. To test for this, we reran our analysis of the mutations detected in the metagenomic sequencing of the patient 1252 isolates (Fig. 6c). Lowering the threshold for mutation calling from 10% to 1% increased the total number of mutations called—possibly by increasing the number of false positives—but did not alter our overall result, consistent with the parabolic fitness landscape (see Fig. S5 in the supplemental material).

Second, our mouse experiments rest on the assumption that a single CFU colonized the mouse. Although this cannot be proved, given the inoculum density, the likelihood of colonization by 2 or more CFU was 0.11%, giving us confidence in single-CFU colonization. We also note that at a higher inoculum density of 27 CFU/mouse, 9 out of 10 mice were colonized, indicating that even at high concentrations where it is very likely that every mouse received at least 1 CFU (probability, ∼100%), colonization was not guaranteed in every mouse.

Third, our simulation results assumed specific values for population size and mutation rates. Importantly, however, when we varied those values, we found that the main conclusion was robust: the penicillin-binding protein (*pbp*) mutation fixed in the population and did so at about the same point in time, despite changes in population size and changes in mutation rate (Fig. S6a). The rate of diversification within branch 2 (clones containing the *pbp* mutation) was sensitive to changes in population size or mutation rate, as demonstrated by the various numbers of unique clones at the end of a simulation (Fig. S6b). Therefore, for a given population size, there is an optimal mutation rate that yields results that recapitulate the experimental results, and for a different population size, a different mutation rate yields the same results. Population size and mutation rate are related to each other but fluid; fixing one will then fix the other to optimize the results. Based on these results, if we increase the population size from 1,000 to 5,000, the mutation rate should be slightly less than doubled to obtain the same variation at the end of the simulation (Fig. S6b). We tested this by running 50 simulations with a population size of 5,000 and a mutation rate of 0.007 and got results nearly identical to those obtained with our choice of a population size of 1,000 and a mutation rate of 0.004 (Fig. S6c). We chose the lower population size (1,000) to minimize computational time but found that larger populations give the same result when the mutation rate was appropriately adjusted.

Due to the risk of bloodstream infections from the gut carrying high resistance to antibiotics (6, 27), it is critical to understand the *in vivo* evolution of VRE. Moreover, the intestinal microbiome composition can affect tumor responses to anticancer therapy (28–30). Overall, we believe that our results should caution that whole-genome sequencing from single isolates taken at a single point of patient colonization could lead to incorrect interpretations. The rapid evolution of subspecies diversity in VRE may underlie the heterogeneous responses to drug treatment across time and should be taken into account in the rational design of antibiotic therapies.

## MATERIALS AND METHODS

### VRE isolate collection and culturing.

Stool samples were collected from allo-HCT patients in a study that was approved by the Institutional Review Board of Memorial Sloan Kettering Cancer Center. All study patients provided written informed consent for biospecimen collection and analysis. The study was conducted in accordance with the Declaration of Helskinki.

Stool samples were stored in 2-ml freezer vials at −80°C. The stored content was thawed and streaked with an inoculating loop directly onto selective Difco Enterococcosel agar supplemented with 10 μg/ml vancomycin (EAPv). Fecal pellets collected from mice were immediately kept on ice after harvesting and plated on the same day on EAPv. The plates were incubated aerobically for 48 h at 37°C, and 4 to 10 colonies were selected and restreaked. Stocks were made by growing a restreaked colony in 5 ml brain heart infusion liquid medium (BHI) overnight and growing in a 1:20-ml dilution in 20 ml BHI for 6 h at 225 rpm at 37°C. Eight hundred milliliters of the culture was resuspended in 200 ml of 75% glycerol to obtain 4 15% glycerol stocks per isolate, stored at −80°C. Bloodstream isolates were stored by the Memorial Sloan Kettering Cancer Center (MSKCC) microbiology laboratory and restreaked on EAPv as described above for the fecal isolates.

### Mouse husbandry and *in vivo* VRE colonization.

The *in vivo* experiments were performed in compliance with the Memorial Sloan Kettering Cancer Center’s institutional guidelines and were approved by its Institutional Animal Care and Use Committee. Eight- to 10-week-old wild-type female C57BL/6 mice were purchased from The Jackson Laboratory. Mice were housed in sterile cages with irradiated feed and acidified water. Five microliters of BHI was inoculated with a frozen stock overnight, and on the next day a 1:20-ml dilution was incubated for 2.5 h at 225 pm at 37°C to obtain a mid-log culture of ∼6 × 10^8^ CFU/ml. Serial dilutions were performed in phosphate-buffered saline (PBS). Inocula were plated to determine the number of CFU input per mouse. Mice were pretreated with ampicillin at 0.5 g/liter in the drinking water for 3 days to abrogate colonization resistance and orally gavaged at 100 to 200 μl per mouse. Mice were individually housed at the time of inoculation. The number of mice per group that were colonized (out of 5 or 10 mice total) was assessed on day 1 and day 5 postchallenge by serially diluting fecal pellets in PBS and plating on EAPv. Mice in the group that received the lowest inoculum dose were kept to monitor VRE and maintained on ampicillin-treated water (0.5 g/liter, changed every 5 to 7 days). VRE density was measured as described above every other week henceforth.

In our first *in vivo* experiment, gavage of mice with an inoculum of ∼2 CFU/10 mice (200 μl/mouse) resulted in 1 out of 10 mice being infected. For our second experiment, mice were inoculated with 2 CFU/mouse (100 μl/mouse), resulting in 3 out of 10 mice infected (mice 2, 3, and 4). Here, we used an allogeneic, minor MHC antigen-disparate bone marrow transplantation model, transplanting 129S1/SvImJ mouse donor cells into female C57BL/6 recipients (9). Briefly, mice were given a split 1,100-cGy radiation dose and administered 5 × 10^6^ bone marrow cells via tail vein injection. Mice were then infected with VRE by oral gavage on the same day. A similar final experiment was performed with a low-dose inoculum from a laboratory strain of E. faecium ATCC 700221. Mice were housed singly once they were inoculated with VRE. When we inoculated 10 mice with the pt110 isolate inoculum at a concentration of 0.2 CFU/mouse, 1 mouse was colonized. Assuming a Poisson distribution, the probability that the mouse was colonized with 2 or more CFU was 0.0011, giving us confidence that it was colonized with only 1 CFU. (The probability that a mouse with this inoculation density would be colonized by exactly 1 CFU is 0.16, and the probability that a mouse with this inoculation density would be colonized by 0 CFU is 0.82.) We also found that even at a much higher inoculum density (27 CFU/mouse), 9 out of 10 mice were colonized, indicating that even at high concentrations, where it is very likely that every mouse received at least 1 CFU (probability ∼1), colonization was not guaranteed in every mouse.

A similar experiment with the ATCC strain inoculum confirmed these results: 3.2 ± 2.8 CFU/mouse led to the colonization of 1 mouse. At the lower end of this range, the probability that the mouse was colonized with 2 or more CFU was 0.008. Likewise, at a much higher inoculum density (72 ± 10.6 CFU/mouse), not every mouse was colonized, even though it is very likely that every mouse received at least 1 CFU (probability, ∼1). There was some variation in the number of CFU required for establishment of VRE, depending on the inoculum used, although the range was similar for both. (The above-described probability calculations were done using the *poisspdf* and *poisscdf* functions in Matlab software.)

### Whole-genome sequencing, reference assembly, and annotation.

Isolates were grown in BHI until early stationary phase and centrifuged. As previously described, DNA was extracted using phenol-chloroform extraction with bead beating using 0.1-mm zirconia-silica beads (BioSpec Products) (6). After extraction, DNA was precipitated in ethanol, resuspended in Tris-EDTA (TE) buffer with 200 μg/ml RNase, and further purified with QIAamp mini-spin columns (Qiagen). The amplicons were purified with a Qiagen QIAamp kit (Qiagen). The purified PCR products were quantified. Purified DNA was sheared using a Covaris ultrasonicator, and size selection was performed with AMPure XP beads (Beckman Coulter). Libraries were prepared for Illumina MiSeq sequencing with a Kapa library preparation kit with Illumina TruSeq adaptors to create 2 × 300-bp paired-end reads according to the manufacturer’s instructions. Fecal pellets sent for shotgun metagenomics sequencing were sequenced with an Illumina HiSeq platform by 2 × 100-bp paired-end reads. Three fecal VRE isolates from pt110 were sequenced at the Broad Institute on the Illumina HiSeq platform by 2 × 101-bp paired-end reads (36).

Sequencing reads were processed using a custom bash shell script (available in the supplemental material as trim.sh files). Trimmomatic (version 0.36) was used to quality filter raw sequence reads. Read quality was assessed by use of the FastQC (version 0.11.5) program.

For pt110, PacBio reads were assembled by use of the hierarchical genome assembly process (HGAP) and iteratively corrected using the Pilon program with Illumina MiSeq reads from the same isolate extracted on day 8. A consensus sequence was constructed using the gatk (version 3.6) and picard (version 2.12.1) programs for an isolate from day −6 using *breseq* SNV and indel calls to correct the PacBio reference sequence. For all other patients, a hybrid assembly was constructed with Oxford Nanopore MinION and MiSeq reads with the Unicycler program and iteratively corrected with the *breseq* (version 0.31.1) tool. Contigs of less than 500 bp were removed postassembly. Quality assessment of finished assemblies was performed using the QUAST (version v4.5) tool. References were annotated using the PATRIC web portal. Chromosomal contigs were determined by BLAST analysis of the assembly fasta file (https://blast.ncbi.nlm.nih.gov/) against the nucleotide collection (nr/nt database) for Enterococcus faecium.

### Variant detection.

Trimmed quality-filtered Illumina reads were aligned to our in-house references, and variants were called using the *breseq* (version 0.31.1) tool. For the isolates, the consensus mode was used. Metagenomes obtained by shotgun sequencing were run in the polymorphism mode. The *breseq* tool’s annotated output files were parsed and analyzed in custom Python scripts (available in the files in the supplemental material). In downstream analyses, we used the *breseq* tool’s default setting frequency cutoff, which called SNVs at a 5% frequency. This threshold was lowered to a 1% frequency for the experiment whose results are shown in Fig. 5f. For pt1252 isolate shotgun sequencing variant analysis, stool samples with >90% VRE domination were analyzed and SNVs were called along 5 chromosomal contigs (contigs 1 to 4 and 8).

For samples analyzed by shotgun sequencing, genes with >15 SNVs per coding sequence were removed as outliers. Select regions were visually inspected in the Integrative Genomics Viewer (version 2.3.97), and for some genes, SNVs were colocalized on the same mapped reads (while other reads had no SNVs), suggesting that these variants were acquired together and did not represent individual mutation events. Other hot spot genes had mapping qualities of ≤2, indicating that poorly mapped DNA might have come from a different bacterial source in the gastrointestinal tract or from another location in the E. faecium genome. Taking a conservative approach, these genes were removed from downstream analysis (*n* = 133 genes from mouse 5, *n* = 91 genes from mouse 1, and *n* = 34 genes from mouse 2). Removing these genes did not alter the shape of the total SNV accumulation over time in our mouse experiments (see Fig. S4a to e, columns 2 and 3, in the supplemental material).

To detect mutations that occurred independently in our *in vivo* experiments, we generated a list of chromosomal SNVs for each mouse that occurred at any time point in the shotgun sequencing data. We compared each position and report the SNVs that occurred in the same position in ≥2 mice in the pt110 isolate-inoculated mice.

### Mathematical modeling.

Genotypes were determined based on coexisting mutation patterns in mouse isolates. These genotypes informed the phylogenetic graphs that were created using custom Matlab scripts (Fig. 4a). (Similar graphs were created for all mice for which the results are presented in Fig. 5 in order to create the fishplots depicted, but these are not shown.) VRE genotypes were plotted by day using isolates alone (Fig. 4b) as well as estimated frequencies from shotgun sequencing data (Fig. S2a) using custom Matlab scripts. Fishplots were generated in R as described previously (31) (Fig. 4c; Fig. 5a to d, column 1; Fig. S2b).

*In silico* experiments were conducted using custom Matlab scripts based on principles from the quasispecies model (32): we modeled a group of related genotypes with offspring that accumulate mutations relative to the sequence of the parent. Our simulations also maintained the assumptions of the quasispecies model, incorporated into a stochastic model. An arbitrary population size of 1,000 was chosen, with all members initially being the ancestral clone. Based on data from mouse 1, over the course of the experiment there were 25 loci that had mutations; therefore, our model also allowed mutation in any of 25 sites. There were 2^25^ possible genotypes, and the fitness of each was set to 0.001 (an arbitrary low number), thus giving a low fitness to clones that were not experimentally observed throughout the duration of the simulation. Under the neutral evolution condition, further fitness enhancement was not done.

While under neutral fitness all clones are given the same fitness, in the other fitness landscapes, clones that acquire mutations are given altered fitness if the mutations match those that were experimentally observed. In the case of a linear fitness landscape, the fitness *f* of genotypes experimentally observed was *f* = (0.1 × number of mutations × 2) + 1. In the case of a logarithmic fitness landscape, the fitness *f* of genotypes experimentally observed was *f* = log(number of mutations + 2). In the case of a parabolic fitness landscape, the fitness *f* of genotypes experimentally observed was *f* = −0.0625 × (number of mutations + 1)^2^ + 0.5 × (number of mutations + 1) + 0.1.

These formulae were determined empirically by determining which parameters most closely recaptured the experimental data in the test simulations. For example, many possible linear formulae with various steepnesses of the slope were tested, and simulation results were compared to the experimental data. After many such trials, the formula for a linear landscape that best represented the experimental data was selected as the best-case scenario for a linear landscape. The same was done for the other fitness landscapes tested, and the best cases for each were compared to each other. Conditions were also tested where clones with the penicillin-binding protein mutation gained an additional fitness advantage, mathematically incorporated by adding (+1) to the formulae presented above. During each round of the simulation, certain members of the population were randomly selected to divide, with genotypes with a higher fitness being more likely to be selected. Each of these selected individuals was then allowed to randomly mutate any of the 25 loci. These new offspring were then added back to the population. To keep the total population constant, random individuals were removed. This was repeated 5,000 to 8,000 times. The condition was also tested where if, during the course of random mutation an individual acquired the *pbp* mutation, that offspring now had the fitness conferred by either the linear, log, or parabolic landscape being tested, regardless of whether that genotype was experimentally observed. The outcome of the above-described *in silico* experiments showed a simulated VRE evolution over time under various possible fitness landscapes (Fig. 4d and e; Fig. S2c to f).

Determining the fitness landscape that best captured the experimental data was done by (i) comparing the time to fixation of the *pbp* mutation in *in vivo* and *in silico* experiments and (ii) calculating how well the scale of time in terms of days for the experiments matched the scale of time in terms of the number of iterations for the simulations. This was calculated as follows.

In the experiment, two major events occur: (i) a decline of the ancestral clone and (ii) expansion of branch 2 (*pbp*-containing clones). For the experimental data, we calculated the rates of decline and expansion, which we call *K_a_* and *K_b_*, respectively. For each fitness landscape, we also calculated *K_a_* and *K_b_* based on the simulation results. The ratio between *K* values was calculated, such that α_1_ = *K_a_*(simulated)/*K_a_*(experimental) and α_2_ = *K_b_*(simulated)/*K_b_*(experimental). Each alpha value has the units number of days per iteration, giving a scaling between experimental days and simulation iterations. Closely matching α_1_ and α_2_ values indicated consistent population dynamics between experimental and simulated data. Of the 6 fitness landscapes tested, only 2 had α_1_ and α_2_ values that were close in value, indicating that the simulation accurately captured both the rate of ancestral decline and the rate of *pbp* clone expansion in the same simulation. These 2 landscapes (linear and parabolic with a boost to *pbp* mutation) were therefore the ones considered top hits, with parabolic being the best match, given the rate of fixation of the *pbp* mutation. The average between α_1_ and α_2_ for the linear landscape led to the 60-iterations/day scaling for linear, and the average between α_1_ and α_2_ for the parabolic landscape led to the 50-iterations/day scaling for parabolic.

### Construction of phylogenetic trees.

Trimmed reads were assembled into contigs using the short-read genome assembler SPAdes (version 3.11.1) and annotated with the PROKKA (version 1.12) software tool. Strains were typed by MLST using the mlst (version 2.8) tool. Core genomes and accessory genes were identified by the Roary (version 3.8.2) program, and SNVs were called with the SNV-sites (version 2.3.2) program (33). Maximum likelihood trees based on SNVs in the core genomes were created with the RAxML program using the model ASC-GTRGAMMA for trees with less than 50 genomes. The phylogenetic tree of the patient VRE strains was created using the FastTree program, which infers the approximately maximum likelihood tree from alignments of sequences (34, 35). The tree was then rooted by the use of Enterococcus hirae as the outgroup.

## Supplementary Material

Supplemental file 1
