# Supplementary material for: Diversification and Evolution of Vancomycin-Resistant Enterococcus faecium during Intestinal Domination
Source: Infect Immun. 2019 Jun 20;87(7):e00102-19. doi: 10.1128/IAI.00102-19 (PMC6589067; doi:10.1128/IAI.00102-19)
Supplement: Supplemental file 1 [file IAI.00102-19-s0001.pdf]

Supplementary Figure Legends

Supplementary Figure 1 | Maps of the variants found in the chromosome across isolates from each MLST. Dark blue squares indicate a variant relative to the reference strain. The starred plot corresponded to the starred sub-branch in Figure 2e.

Supplementary Figure 2 | a) The frequency for each mutation found in the isolate collection from Figure 4b was estimated by shotgun sequencing, and the proportions of related strains are plotted over time. **b)** Clonal dynamics are captured in a fishplot. **c)** The relative fitness of each clone is plotted over time under linear, log, and parabolic fitness landscapes according to the number of mutations per clone (solid lines). Dotted lines depict the higher "boosted" fitness landscapes granted to clones with a *pbp5* mutation under certain simulation conditions. **d)** Simulated VRE evolution under a linear fitness landscape with no additional boost given to experimentally observed genotypes with a *pbp5* mutation. Each genotype corresponds to a color, the same scheme used in Fig 4eOne "day" corresponds to about 60 simulation iterations. **e-f)** Simulations were run in which any clone with a *pbp5* mutation was given a fitness according to the landscapes defined in Supplementary Figure 3c, regardless of whether the clone was experimentally observed (pink) or in which only experimentally observed clones were given this fitness landscape (blue). At the end of the simulation, the percent of clones harboring a *pbp5* mutation that were also experimentally observed was calculated; this was repeated 50 times and plotted as a bar and whiskers plot with "+" marking outliers. Increasing fitness boosts to branch 2 were also tested. These simulations were done for linear (e) and parabolic (f) fitness landscapes.

Supplementary Figure 3 | Maps of the variants found in the chromosome across isolates from each mouse. Dark blue squares indicate a variant relative to the reference strain.

Supplementary Figure 4 | From left to right: Frequency traces for chromosomal mutations picked up by shotgun sequencing of fecal pellets as done in Figure 3e; Total number of mutations that achieved ≥5% frequency in the shotgun data per day over the course of the experiment; Total number of mutations with no frequency cutoff in the shotgun data per day over the course of the experiment; The average frequency of variants per day in shotgun vs. isolate data for each mouse.

Supplementary Figure 5 | The number and cumulative number of variants per day from the data in Figure 6c, re-analyzed with a range of thresholds for mutation calling.

Supplementary Figure 6 | a) The time it took for the *pbp5* mutation to fix in the population (in terms of number of iterations) is plotted against mutation rate, for several population sizes simulated. b) The number of unique clones at the end of a simulation was calculated for several population sizes and mutation rates. c) Simulated VRE evolution under a parabolic fitness landscape with a fitness boost given to experimentally observed genotypes with a *pbp5* mutation. Population size is 5000 and mutation rate is .007. Color scheme and scaling are identical to that in figure 4e.

Supplementary Table 1 | dN/dS analysis of patient data, looking at recurrent mutations across patient isolates.

Supplementary Table 2 | A table of mutations found in 3 or more isolates from the experiment in Figure 3. The second column indicates the number of isolates that carry that mutation.

Supplementary Table 3 | Table of mutations found in more than one mouse or more than one locus per gene.

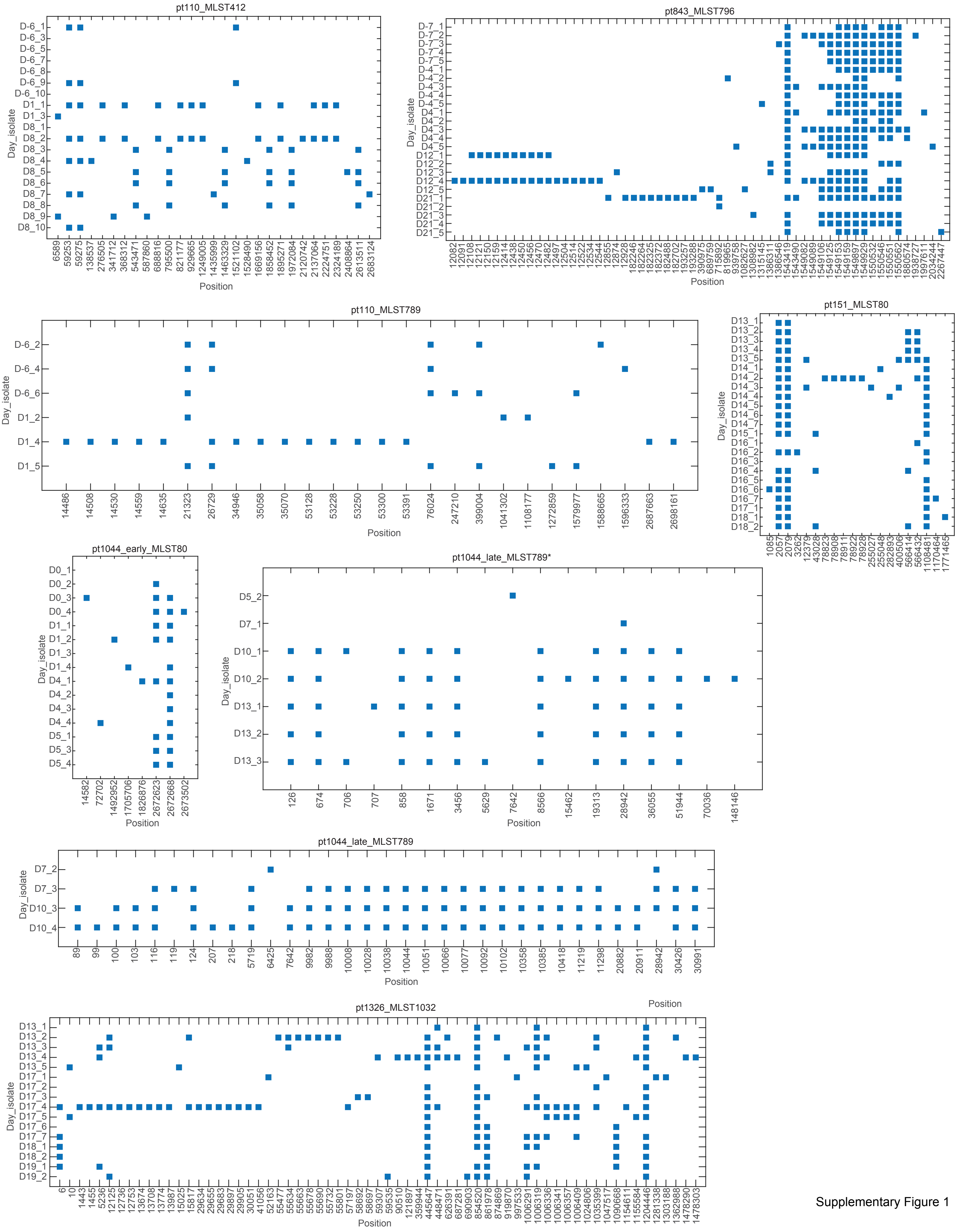

Supplementary Figure 1

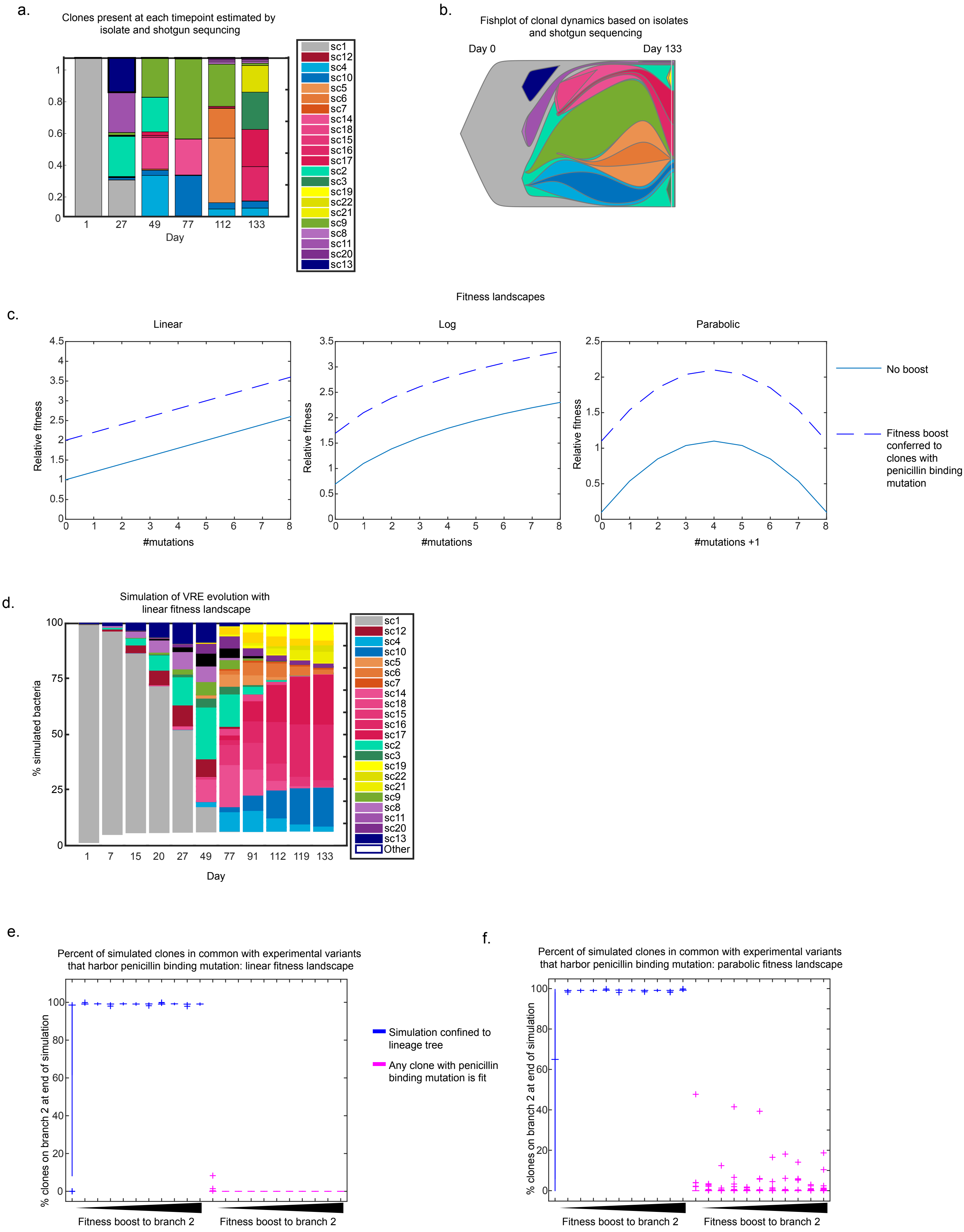

Supplementary Figure 2

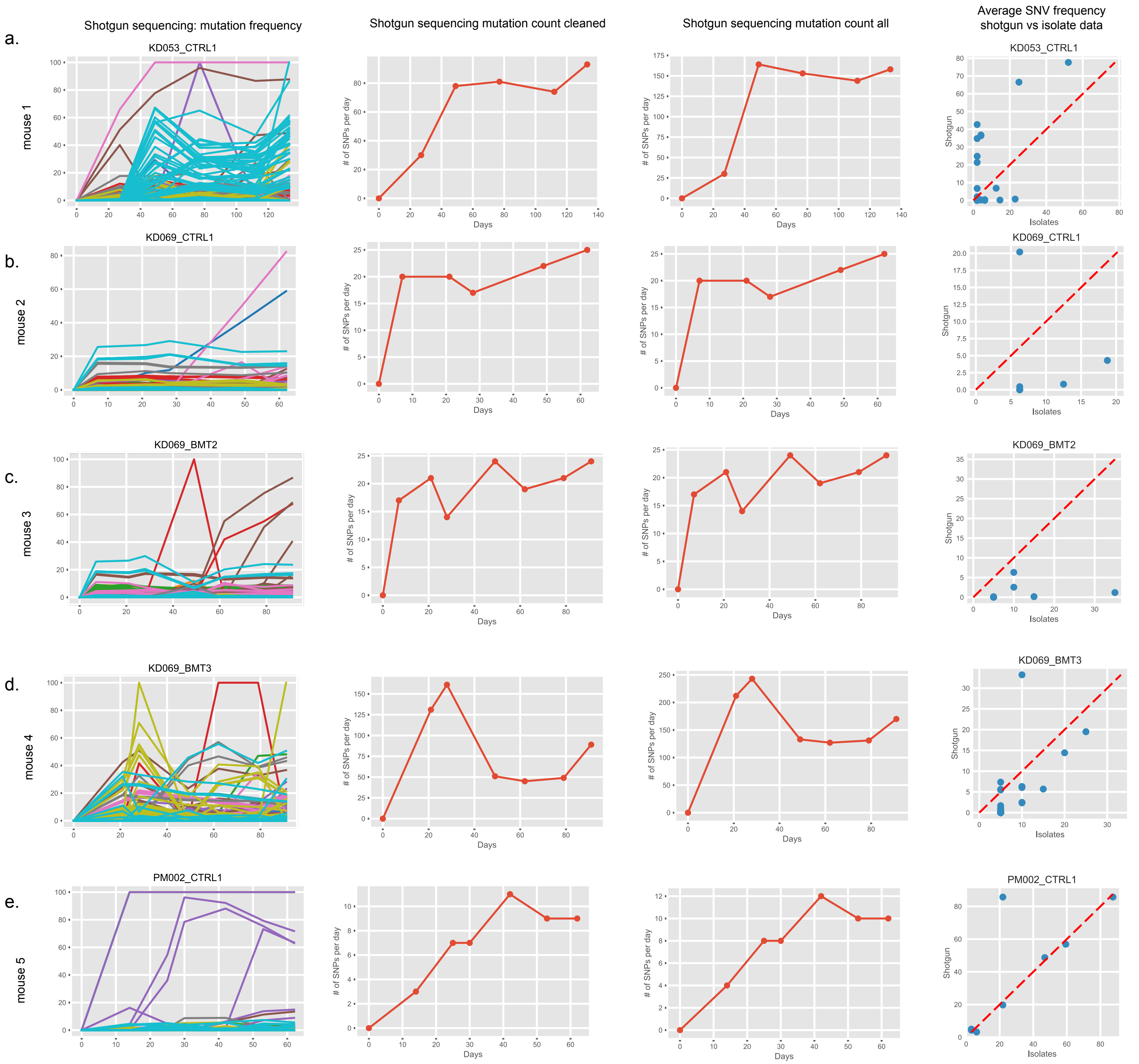

Supplementary Figure 4

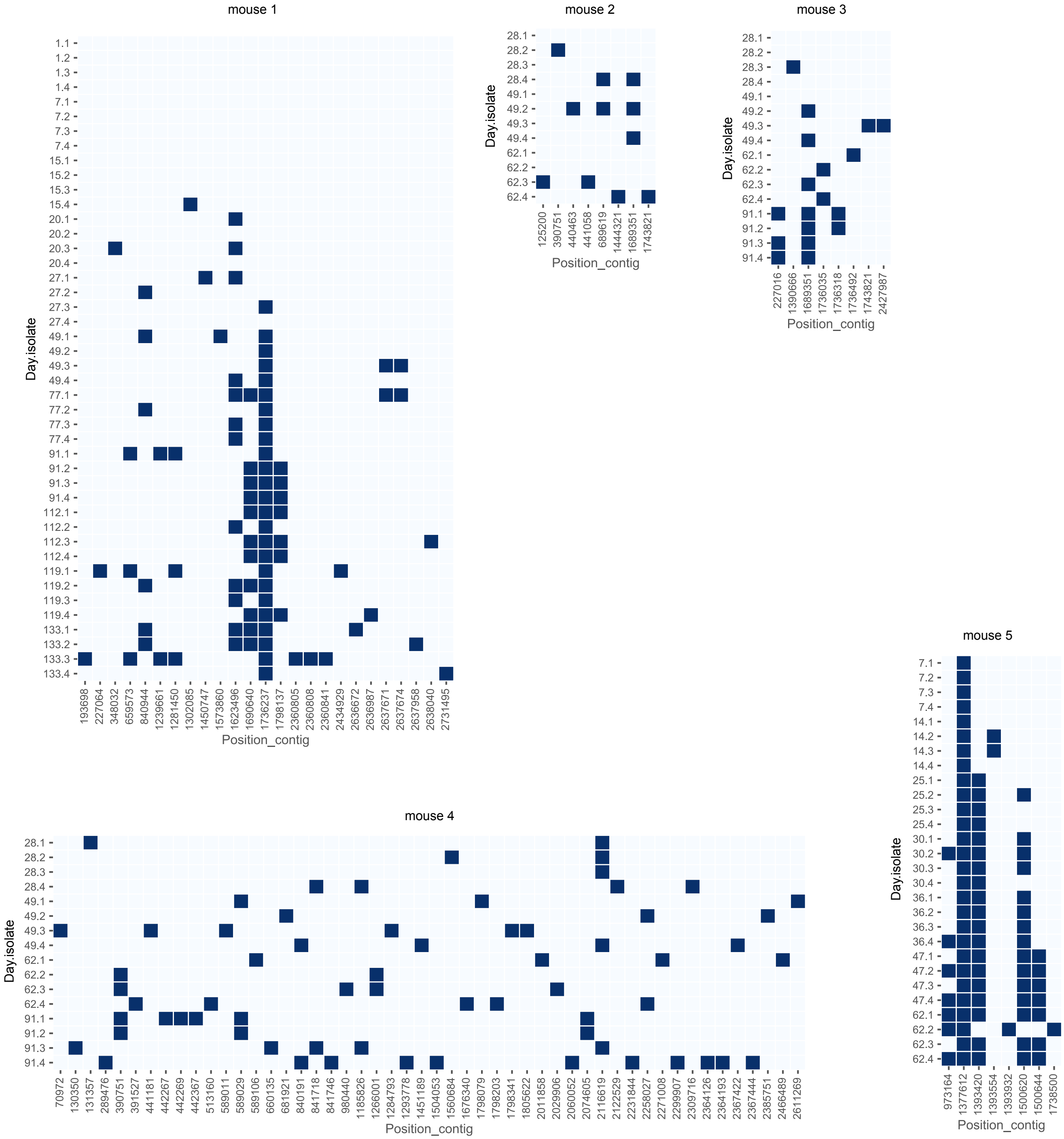

Supplementary Figure 3

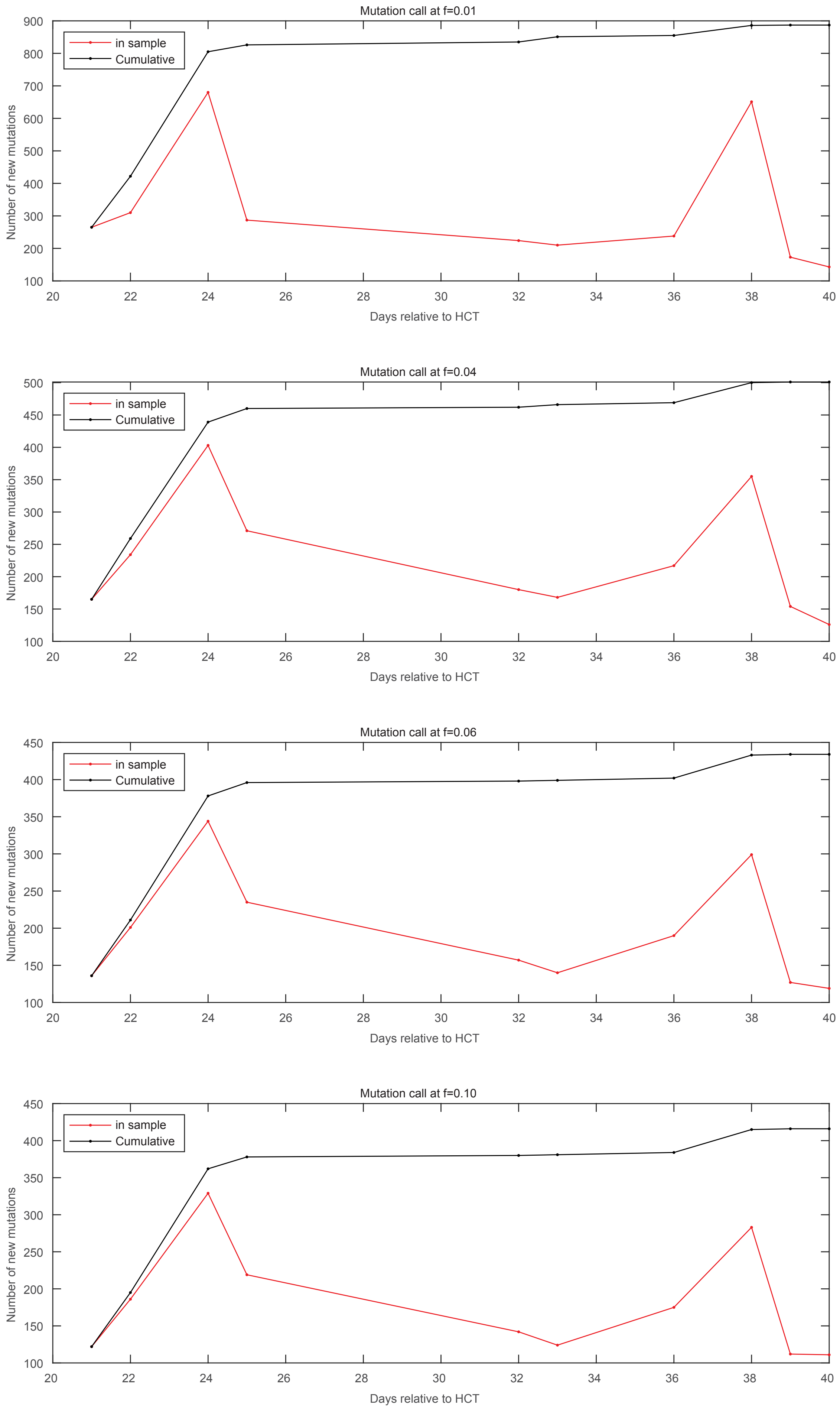

Supplementary Figure 5

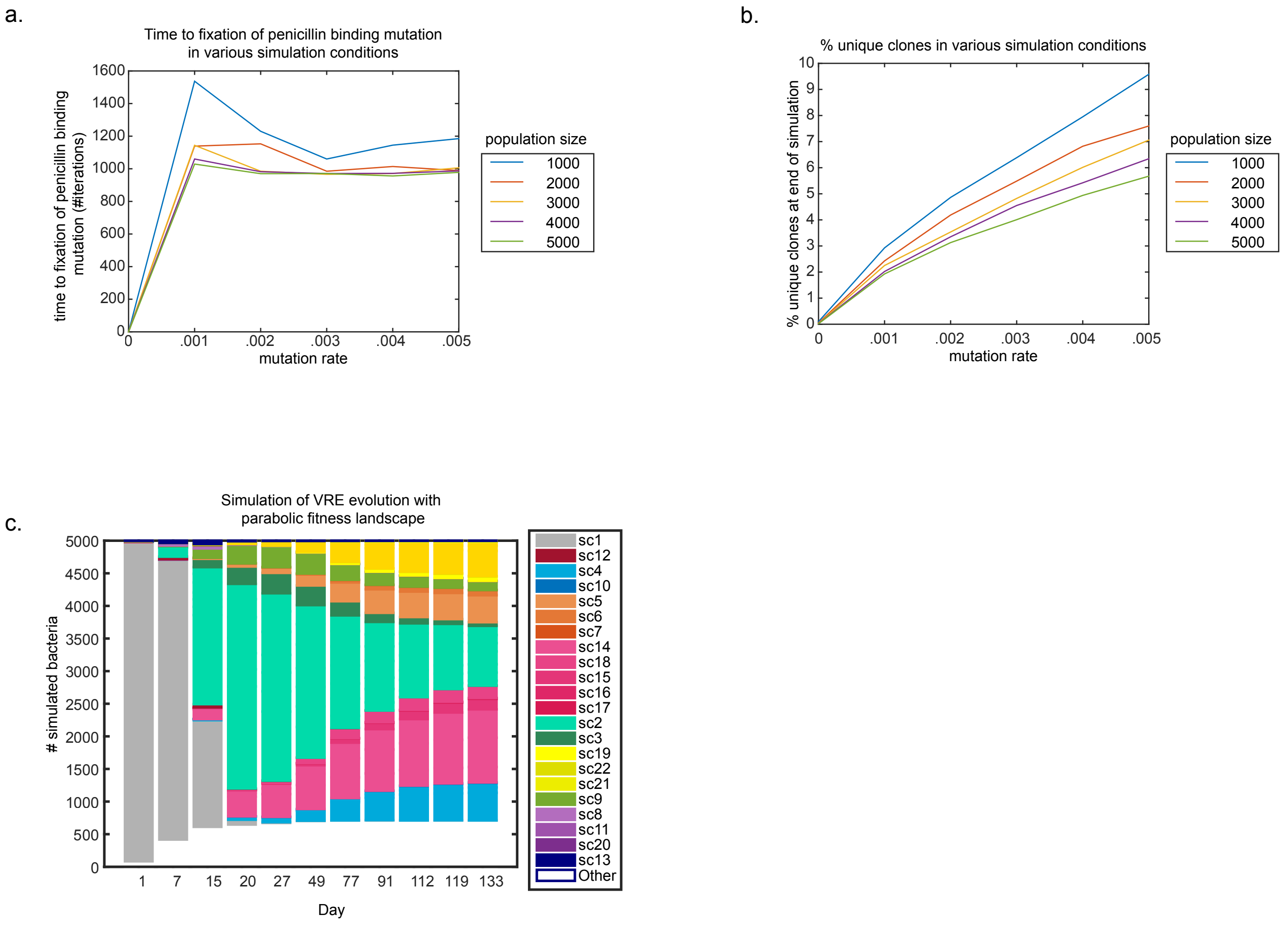
a.
Time to fixation of penicillin binding mutation
in various simulation conditions
time to fixation of penicillin binding
mutation (#iterations)
0
200
400
600
800
1000
1200
1400
1600
0
.001
.002
.003
.004
.005
mutation rate
population size
1000
2000
3000
4000
5000
b.
% unique clones in various simulation conditions
% unique clones at end of simulation
0
1
2
3
4
5
6
7
8
9
10
0
.001
.002
.003
.004
.005
mutation rate
population size
1000
2000
3000
4000
5000
c.
Simulation of VRE evolution with
parabolic fitness landscape
simulated bacteria
0
500
1000
1500
2000
2500
3000
3500
4000
4500
5000
1
7
15
20
27
49
77
91
112
119
133
Day
sc1
sc12
sc4
sc10
sc5
sc6
sc7
sc14
sc18
sc15
sc16
sc17
sc2
sc3
sc19
sc22
sc21
sc9
sc8
sc11
sc20
sc13
Other

dN/dS analysis of patient data

Function	dN	dS	dN/dS
'FIG00629435: hypothetical protein'	7	7	1
'FIG00630388: hypothetical protein'	4	0	Inf
'Surface protein Rib'	3	0	Inf
'FIG00630166: hypothetical protein'	2	1	2
'NtrC family Transcriptional regulator, ATPase domain'	2	0	Inf
'D-alanine--D-alanine ligase (EC 6.3.2.4)'	2	0	Inf
'Sorbitol operon transcription regulator'	2	0	Inf
'FIG00628756: hypothetical protein'	2	2	1
'MSM (multiple sugar metabolism) operon regulatory protein'	2	0	Inf
'23S rRNA (adenine(2058)-N(6))-dimethyltransferase (EC 2.1.1.184) '	2	0	Inf
'FIG00628730: hypothetical protein'	1	2	0.5
'FIG00628828: hypothetical protein'	1	1	1
'Sortase A, LPXTG specific'	1	1	1
'FIG00627764: hypothetical protein'	1	1	1
'FIG00630096: hypothetical protein'	0	2	0
'FIG00629366: hypothetical protein'	0	2	0

Position	No.	Product
1736237	25	Penicillin-binding protein 5 (pbp5)
1623496	12	LytR transcriptional regulator (hypothetical)
1690640	11	PTS system, gluconate-specific IIC/D component
1798137	7	Conserved hypothetical protein
840944	6	Two-component sensor kinase SA14-24
659573	3	Transcriptional antiterminator of lichenan operon (licR)
1281450	3	D-glycero-beta-D-manno-heptose-7-phosphate ($C_7H_{15}O_{10}P$)

Supplementary Table 2

	# Mice	Avg SNPs/gene	VRE strain
Penicillin-binding protein 5	3	2.3	pt110, ATCC
PTS system, gluconate-specific IIC/D component	3	1	pt110 only
Two-component sensor kinase SA14-24	2	1.5	pt110 only
*Lactose PTS system repressor	2	1	pt110 only
ABC transporter (maltose/g3p/polyamine/iron)	1	2	ATCC only

*Note: IIC in KD069 ctrl1 (ms #3) and BMT2 (ms #4), IID in KD053 (ms #1)

Supplementary Table 3
